# Loss of complement regulatory proteins on red blood cells in mild malarial anaemia and in *Plasmodium falciparum* induced blood-stage infection

**DOI:** 10.1186/s12936-019-2962-0

**Published:** 2019-09-18

**Authors:** Damian A. Oyong, Jessica R. Loughland, Arya SheelaNair, Dean Andrew, Fabian D. L. Rivera, Kim A. Piera, Timothy William, Matthew J. Grigg, Bridget E. Barber, Ashraful Haque, Christian R. Engwerda, James S. McCarthy, Nicholas M. Anstey, Michelle J. Boyle

**Affiliations:** 1 Menzies School of Health Research, Darwin, NT Australia; 2 Charles Darwin University, Darwin, NT Australia; 3 QIMR Berghofer Medical Research Institute, Brisbane, QLD Australia; 4 Infectious Diseases Society, Sabah-Menzies School of Health Research Clinical Research Unit, Queen Elizabeth Hospital, Kota Kinabalu, Sabah Malaysia; 5 Gleneagles Medical Centre, Kota Kinabalu, Sabah Malaysia; 6 School of Medicine, University of Queensland, Brisbane, QLD Australia; 7 Burnet Institute, Melbourne, VIC Australia

**Keywords:** Malaria, Anaemia, Complement, Complement regulatory proteins, *falciparum*, *vivax*

## Abstract

**Background:**

Anaemia is a major consequence of malaria, caused by the removal of both infected and uninfected red blood cells (RBCs) from the circulation. Complement activation and reduced expression of complement regulatory proteins (CRPs) on RBCs are an important pathogenic mechanism in severe malarial anaemia in both *Plasmodium falciparum* and *Plasmodium vivax* infection. However, little is known about loss of CRPs on RBCs during mild malarial anaemia and in low-density infection.

**Methods:**

The expression of CRP CR1, CD55, CD59, and the phagocytic regulator CD47, on uninfected normocytes and reticulocytes were assessed in individuals from two study populations: (1) *P. falciparum* and *P. vivax*-infected patients from a low transmission setting in Sabah, Malaysia; and, (2) malaria-naïve volunteers undergoing *P. falciparum* induced blood-stage malaria (IBSM). For clinical infections, individuals were categorized into anaemia severity categories based on haemoglobin levels. For IBSM, associations between CRPs and haemoglobin level were investigated.

**Results:**

CRP expression on RBC was lower in Malaysian individuals with *P. falciparum* and *P. vivax* mild malarial anaemia compared to healthy controls. CRP expression was also reduced on RBCs from volunteers during IBSM. Reduction occurred on normocytes and reticulocytes. However, there was no significant association between reduced CRPs and haemoglobin during IBSM.

**Conclusions:**

Removal of CRPs occurs on both RBCs and reticulocytes during *Plasmodium* infection even in mild malarial anaemia and at low levels of parasitaemia.

## Background

Human infection with *Plasmodium* species is often complicated by anaemia, which is a major contributor to morbidity and mortality in malaria endemic regions [1, 2]. Young children and pregnant women typically suffer the most from malarial anaemia [3–5]. The pathophysiology of malarial anaemia is multifactorial [6] with reduction in haemoglobin largely driven by loss of uninfected red blood cells (RBCs) [7, 8]. Different overlapping mechanisms are involved in the progression of malarial anaemia, including bone marrow dysfunction and dyserythropoiesis, splenic retention of RBCs, and complement-mediated destruction of RBCs [2, 9, 10]. Destruction of RBCs by complement attack is mediated through the removal of complement regulatory proteins (CRPs) on the RBC surface and consequent complement deposition [11–13].

Activation of the complement system can occur under the classical, alternative, and lectin cascade pathway, all of which are regulated by CRPs. Membrane-bound CRPs are expressed on the surface of cells, including RBC, and serve to protect from complement attack [14]. Three different CRPs are expressed on RBC surface: complement receptor 1 (CR1/CD35), decay-accelerating factor (DAF/CD55), and protectin (CD59). Membrane-bound CRPs can be removed during *Plasmodium* infection through the binding of antigen–antibody complexes to RBC CRPs [12, 13, 15, 16]. Together, the whole complexes are transported by macrophages to the liver and spleen for removal while RBCs, striped of CRPs, are recirculated. After repeated cycles, the CRP-deficient RBC becomes susceptible to C3b deposition and destruction by macrophages [17]. Macrophage-mediated erythrophagocytosis is also regulated by the self-marker protein CD47, expressed on the RBC surface [18]. A recent study shown that the loss of CRPs during malaria is mainly restricted to uninfected RBCs, rather than parasitized RBCs [19].

Significant reductions of CR1 and CD55 have largely been reported in patients with severe anaemia from *Plasmodium falciparum* infection [11, 13, 20], and more recently from *Plasmodium vivax* infection [19]. One study also showed reduction of CD55 in anaemic children with haemoglobin < 10 g/L, which includes mild and severe anaemia cases. However, most previous studies have focused on the loss of CRPs on RBC during severe malarial anaemia, and the role of RBC CRP removal in the substantial burden of mild but often chronic anaemia in low transmission settings is not fully elucidated. Further, whether RBC CRPs are also lost prior to the onset of anaemia during low density infections is yet to be evaluated. Here, the reduction of CRPs was investigated in two settings, *P. falciparum* and *P. vivax*-infected patients from low transmission settings of Sabah, Malaysia and malaria naive volunteers undergoing *P. falciparum* induced blood-stage malaria (IBSM).

## Methods

### Ethics statement

Written informed consent was obtained from all study participants, with consent obtained from parents or guardians in the case of children enrolled in the Malaysian studies. For clinical cohorts, sample collection was approved by the Ethics Committee of Menzies School of Health Research, Darwin, Australia and from Medical Research and Ethics Committee, Ministry of Health Malaysia. The IBSM trial was approved by QIMR Berghofer Ethics Committee, Brisbane, Australia.

### Study cohort

#### Sabah, Malaysia

Patients with malaria were enrolled from an observational study conducted in Sabah, Malaysia, described previously [21]. Briefly, patients with positive malaria by microscopy detection were enrolled if they were non-pregnant, ≥ 12 years old, within 18 h of commencing malaria treatment, and had no major comorbidities or concurrent illness. Infection status was later confirmed using polymerase chain reaction (PCR), and mixed-species or parasite negative infection were retrospectively excluded. In this study, samples were used from patients with PCR-confirmed falciparum and vivax malaria. Patients were treated using hospital guidelines with chloroquine-primaquine or artemisinin combination therapy (ACT). Samples were also obtained from healthy controls, who were visitors or patients’ relatives with no history of fever in the last 48 h and who were blood film negative by microscopy and were confirmed negative to *Plasmodium* infection by PCR.

Glycerol-preserved RBC samples were obtained from 24 individuals (n, *P. falciparum* = 10, *P. vivax* = 10, healthy control = 4). Samples were selected based on availability. Selected samples were grouped into anaemia categories based on World Health Organization (WHO) recommendation [22]. Non-anaemia was defined as haemoglobin levels higher than 130 g/L for men and 120 g/L for women. Mild-anaemia was defined as haemoglobin levels between 80 and 129 g/L for men and 80–119 g/L for women.

#### *Plasmodium falciparum* induced blood-stage malaria (IBSM) trial

The administration of blood-stage parasites to healthy volunteers, including inoculum preparation, volunteer recruitment, viable parasite quantification, monitoring and treatment were performed as previously described [23]. Briefly, healthy malaria-naïve volunteers were inoculated with 2800 viable *P. falciparum* 3D7-parasitized RBCs, and parasitaemia was quantified daily by qPCR. Participants were treated with anti-malarial medication at day 8 of infection. For this study, glycerol-preserved RBCs samples were also collected from participants enrolled in a *P. falciparum* IBSM study in Brisbane, Australia. The IBSM study trial was registered at NIH ClinicalTrials.gov, NCT03542149. For the purpose of the current sub-study, blood from 16 healthy malaria-naive volunteers (18-55 years old) was collected at day 0 prior to infection (defined as day 0 in analysis), and immediately prior to treatment (day 8), and 10, 12, 13, 15, 17/18, and 45 days after inoculation from two cohorts in one infection study. Haemoglobin was measured at days 0, 8, 11, 22, 29, 36, 42, and 45.

### Measurement of CR1, CD55, CD59, and CD47 on RBCs surface

**Fig. 1:**
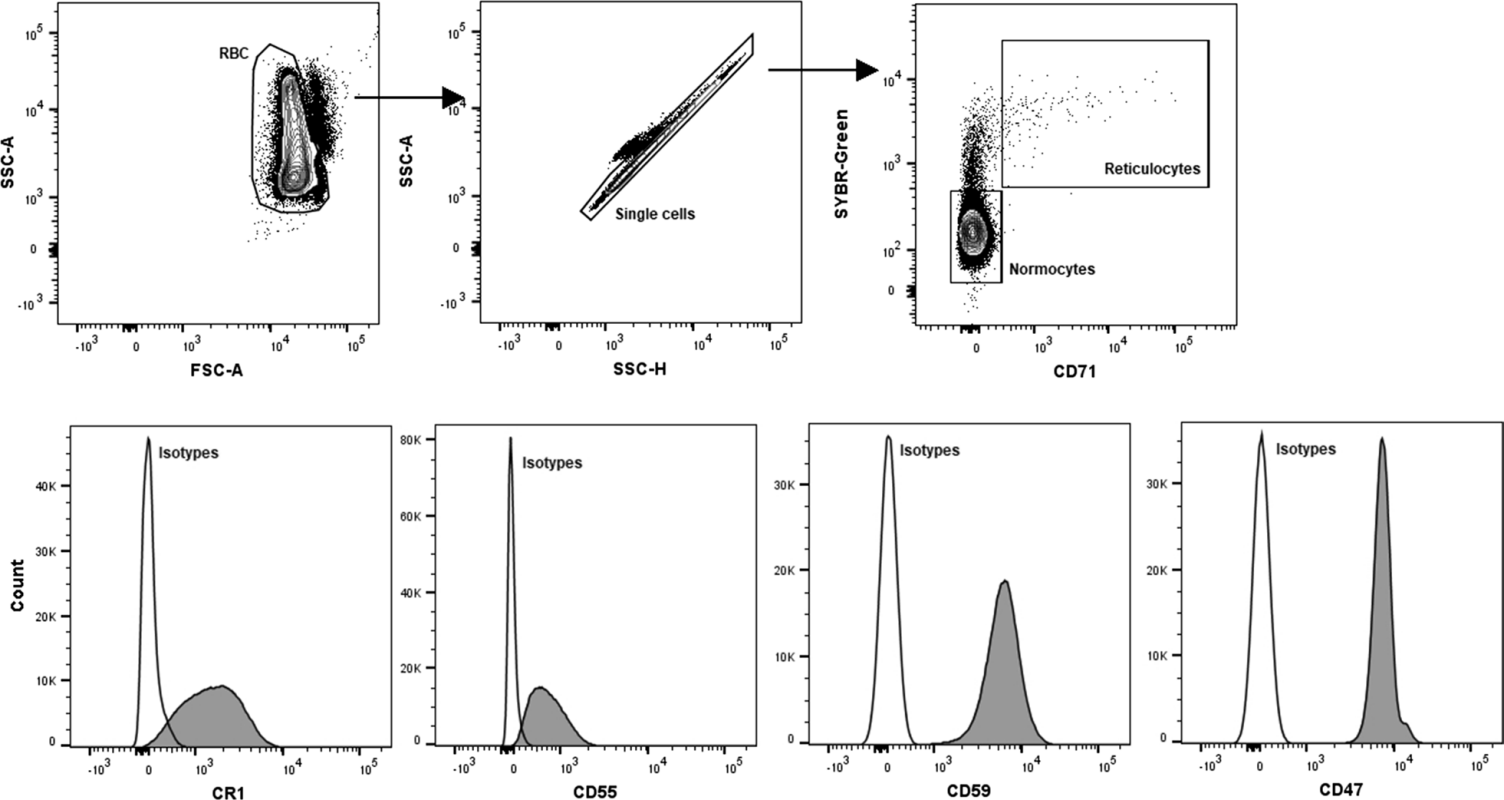
Gating strategy for CRP analysis. RBC was characterized into uninfected normocytes and reticulocytes using SYBR-Green and CD71. CRP expression was measured with anti-CR1, -CD55, -CD59 and -CD47 antibodies. Histogram indicates MFI values of CRPs expression

Frozen glycerol-preserved RBCs from patients were thawed using 3.5% NaCl in distilled water and PBS. To account for variation between thawing and staining batches, median fluorescent intensity (MFI) were standardized using RBCs collected from malaria naïve, Australian individuals (n = 3), cryopreserved in multiple aliquots for use across study. Antibody staining of CRPs was done in individual panels with the addition of SYBR-Green for parasite staining and CD71 to distinguish normocytes and immature reticulocytes (Fig. 1). Briefly, 5 µl of blood pellet were stained with the following antibodies: SYBR-Green (1/10,000 dilutions from 10,000× concentrates in DMSO, Thermo Fisher), anti-human CD71 PE-Cy7 (clone CY1G4, Biolegend), anti-human CR1 BV421 (clone E11, Becton Dickinson), anti-human CD55 APC (clone IA10, Becton Dickinson), anti-human CD59 BV421 (clone p282 H19, Becton Dickinson), anti-human CD47 BV421 (clone B6H12, Becton Dickinson), and isotype controls for each antibody (Becton Dickinson). Samples were stained for 25 min in the dark and at room temperature, followed by washing with PBS and acquisition on Gallios (Beckman Coulter) flow cytometer. For IBSM samples, additional anti-human CR1 PE (clone E11, Biolegend) antibody was used and samples were analysed on a BD LSRFortessa™ (BD Bioscience) flow cytometer. Parasitaemia cut-off point was set at 0.1% for infected RBC measurements. Acquisition data was analysed with Kaluza (version 1.3) and FlowJo (version 10).

### Statistics

All analyses were performed in STATA (version 15.0) and GraphPad Prism (version 7.03). Differences in CRPs and CD47 expression were compared using Wilcoxon signed-rank test. For comparisons between anaemia group, Mann–Whitney nonparametric test was used. Spearman’s nonparametric method was used to determine correlations between CRPs and haemoglobin level.

## Results

### Reduced CR1, CD55, and CD59 on uninfected normocytes and reticulocytes in Malaysian malaria patients with mild anaemia

To establish whether changes in CRP expression on RBC and reticulocyte was a feature of mild anaemia during malaria in low transmission settings, RBC samples were examined from *P. falciparum* and *P. vivax*-infected patients living in Sabah, Malaysia. Demographic and clinical characteristics of the study participants from Sabah, Malaysia are summarized in Table 1. Blood samples were available from 24 participants, including 10 with *P. falciparum* infection, 10 with *P. vivax* infection, and 4 uninfected healthy controls.

**Table 1:** Demographic and clinical parameters of participants from Sabah, Malaysia

	*P. falciparum*		*P. vivax*		Uninfected
	Mild anaemia	Non-anaemia	Mild anaemia	Non-anaemia	
Sample size (male/female)	n = 4 (1/3)	n = 6 (5/1)	n = 3 (3/0)	n = 7 (5/2)	n = 4 (3/1)
Haemoglobin g/dL (IQR)	11.7 (10.0–12.3)	14.0 (13.2–14.2)	10.9 (10.3–11.8)	14.1 (12.5–15.0)	16.6 (16.3–17.5)
Age (year)	37 (26–48)	40 (30–54)	25 (21–36)	32 (19–46)	34 (30–34)
Parasite count (iRBC/µL)	740 (220–2100)*	19,700 (6800–33,600)*	3400 (430–15,000)	6100 (370–10,300	N/A

Parasite count was determined using blood smear microscopy. Median and interquartile ranges are indicated. Parasitaemia and age were compared between mildly anaemic and non-anaemic *P. falciparum* and *P. vivax* patients using Mann–Whitney nonparametric test

* p < 0.05

**Fig. 2:**
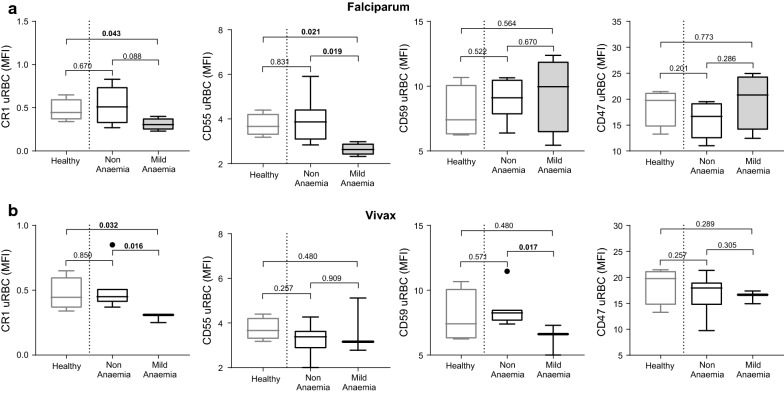
Expression of CR1, CD55, CD59, and CD47 on uninfected normocytes in healthy, non-anaemic, and mildly-anaemic individuals from low malaria endemic region Sabah, Malaysia. CRP and CD47 expression was measured on uninfected normocytes in healthy controls, and patients with uncomplicated falciparum (**a**) or vivax (**b**) malaria. For malaria patients, individuals were grouped as non-anaemic and mild anaemic. p-values indicate Mann–Whitney nonparametric tests between groups. Boxplot’s lower and upper hinges represent first and third quartiles with median line indicated across the box. Whisker lines correspond to highest and lowest values no further than 1.5 interquartile range from the hinges whereas dot points beyond whisker lines are outliers

In *P. falciparum*-infected individuals, CR1 (p = 0.043) and CD55 (p = 0.021) expression on uninfected normocytes was lower in mildly-anaemic patients compared to healthy controls (Fig. 2a). CR1 was also lower on normocytes in mildly-anaemic compared to non-anaemic patients, although this difference was not statistically significant (p = 0.088). CD55 expression on normocytes was significantly lower in infected individuals with mild anaemia compared to healthy controls and to infected but non-anaemic patients (p = 0.021 and p= 0.019 respectively Fig. 2a). Additionally, CR1 and CD55 expression were positively correlated with haemoglobin level on enrolment, albeit only statistically significant for CD55 (CR1, R = 0.62, p = 0.055; CD55, R = 0.68, p = 0.032). No significant differences were observed for CD47 and CD59 expression on normocytes during falciparum malaria.

In vivax malaria, CR1 on uninfected normocytes was lower in mildly anaemic individuals compared to healthy controls and infected non-anaemic individuals (p= 0.032 and p = 0.016 respectively Fig. 2b). CD59 expression on uninfected normocytes was reduced in mildly-anaemic compared to non-anaemic vivax malaria patients (p=0.017, Fig. 2b). CD59 expression was positively associated with haemoglobin level on enrolment (R = 0.64, p = 0.048). Again, no significant differences were observed for CD47 and CD55 expression on uninfected normocytes during vivax malaria.

**Fig. 3:**
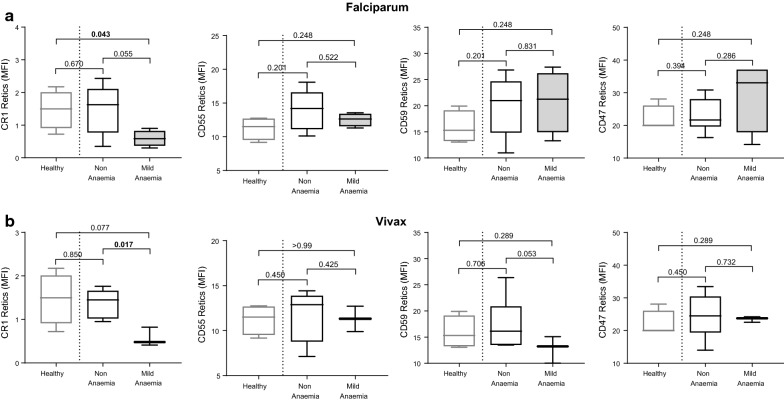
Expression of CR1, CD55, CD59, and CD47 on reticulocytes in healthy, non-anaemic, and mildly anaemic individuals from low malaria endemic region Sabah, Malaysia. CRP and CD47 expression was measured on uninfected reticulocytes in healthy controls, and patients with uncomplicated falciparum (**a**) or vivax (**b**) malaria. For malaria patients, individuals were grouped as non-anaemic and mild anaemic. Boxplot’s lower and upper hinges represent first and third quartiles with median line indicated across the box. Whisker lines correspond to highest and lowest values no further than 1.5 interquartile range from the hinges whereas dot points beyond whisker lines are outliers

**Fig. 4:**
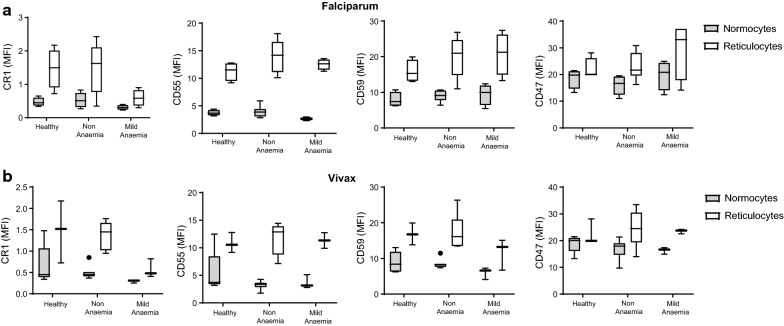
Comparison of CRP expression on uninfected normocytes and reticulocytes population in Sabah patients. CRP and CD47 expression levels, expressed as MFI, in uninfected normocytes and reticulocytes in malaria patients with falciparum (**a**) or vivax (**b**) infection. All comparisons for CRPs expression between normocytes and reticulocytes were statistically significant using Wilcoxon signed-rank nonparametric tests (p < 0.05). Boxplot’s lower and upper hinges represent first and third quartiles with median line indicated across the box. Whisker lines correspond to highest and lowest values no further than 1.5 interquartile range from the hinges whereas dot points beyond whisker lines are outliers

Expression of CR1, CD55, CD59, and CD47 was also assessed on reticulocytes during falciparum and vivax malaria and healthy controls (Fig. 3). There was evidence for reduced levels of CR1 on reticulocytes in infected individuals with mild anaemia in both falciparum and vivax malaria (Fig. 3b). Patients with mild *P. falciparum* and *P. vivax* malarial anaemia also had lower CR1 (p = 0.055) and CD59 (p = 0.053) on reticulocytes compared to non-anaemic malaria patients, respectively (Fig. 3a, b). Further, the level of CD55 and CD59 on reticulocytes was positively associated with haemoglobin level for falciparum and vivax malaria, respectively (CD55: R = 0.53, p = 0.053; CD59: R = 0.55, p = 0.027). Overall, the levels of CRPs on uninfected reticulocytes was higher when compared to uninfected normocytes, including in healthy controls (Fig. 4). No differences were observed for CD47 expression on reticulocytes during falciparum and vivax malaria.

### Transient drop in RBC CRPs following IBSM infection

To understand the dynamics of changes in RBC and reticulocyte CRP expression during malaria, blood samples were examined from volunteers participating in IBSM studies. Demographic and clinical characteristics of the *P. falciparum* IBSM volunteers are summarized in Table 2. Blood samples were available from 16 participants over 45 days following inoculation. Volunteers were treated with anti-parasitic drug at day 8 when parasitaemia reached approximately 20,000 parasite/mL.

**Table 2:** Demographic and clinical parameters of *Plasmodium falciparum* IBSM volunteers

	*P. falciparum* IBSM
Sample size (male/female)	n = 16 (12/4)
Age (year)	22.5 (21.75–28.25)
Parasite count (parasites/mL)	21,626 (6125–41,675)

Data availability	Days												
	0	8	10	11	12	13	15	17	22	29	36	42	45
Haemoglobin	x	x		x					x	x	x	x	x
CRPs	x	x	x		x	x	x	x					x

Parasite count was determined using qPCR. Median and interquartile ranges are indicated

**Fig. 5:**
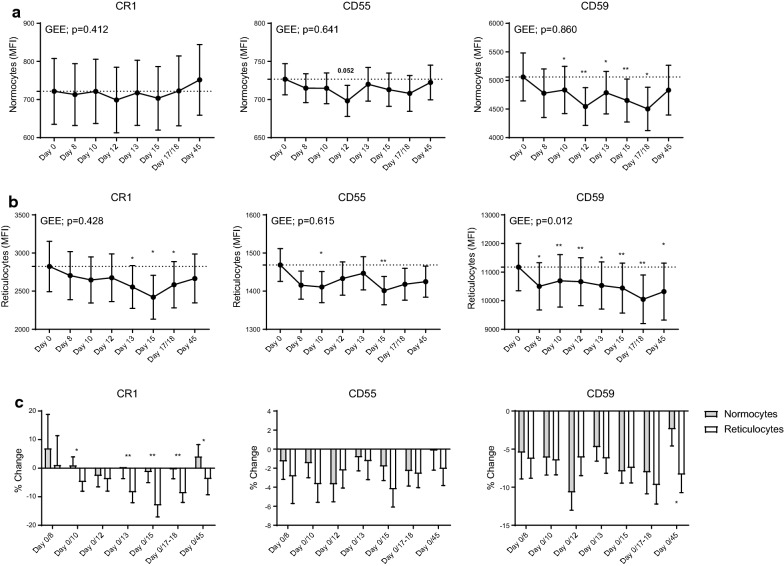
Expression of CR1, CD55, and CD59 on normocytes and reticulocytes in volunteers during induced blood stage *Plasmodium falciparum* infection. Expression of CRPs on uninfected normocytes (**a**) and reticulocytes (**b**) in participants during IBSM. Generalized estimating equation (GEE) and Wilcoxon signed-rank nonparametric tests between each group are indicated. *p < 0.05; **p < 0.01. Data are presented in connecting lines with mean values and error bars indicating standard error. **c** Percentage change of CRPs expression was calculated on MFI change at follow-up visits compared to baseline day 0. Bar chart indicates mean ± standard error. Wilcoxon signed-rank nonparametric tests between each group are indicated. *p < 0.05; **p < 0.01

Expression of CD55 and CD59 on uninfected normocytes was reduced after primary *P. falciparum* infection (Fig. 5a). Compared to day 0, CD55 expression was lower at day 12 (p = 0.05) while CD59 was significantly lower at all time-points except for day 8 and day 45 (Fig. 5a). By day 45, levels of CD55 and CD59 had returned to that of baseline levels. There were no changes in CR1 and CD47 expression on uninfected normocytes over 45 days of follow ups. CRP expression was also assessed on reticulocytes (Fig. 5b). Compared to day 0, CR1 was significantly reduced on days 13 (p = 0.029), 15 (p = 0.011), and 17/18 (p = 0.022), while CD55 was reduced on days 10 (p = 0.039) and 15 (p = 0.005). The expression of CD59 was significantly reduced at all time-points compared to day 0 (Fig. 5b). Again, there were no changes in CD47 expression on reticulocytes over 45 days follow up.

Changes in CRP expression on reticulocytes and RBC normocytes were compared by calculating percentage change from day 0 (before infection). Percentage loss of CR1 was significantly greater in reticulocytes than in uninfected normocytes at all time-points except for day 8 and 12 (Fig. 5c). For CD59, reticulocyte percentage loss was significantly greater at day 45. No significant difference was observed for CD55 percentage loss between normocytes and reticulocytes at all time-points.

### Relationship between haemoglobin and CRPs

**Fig. 6:**
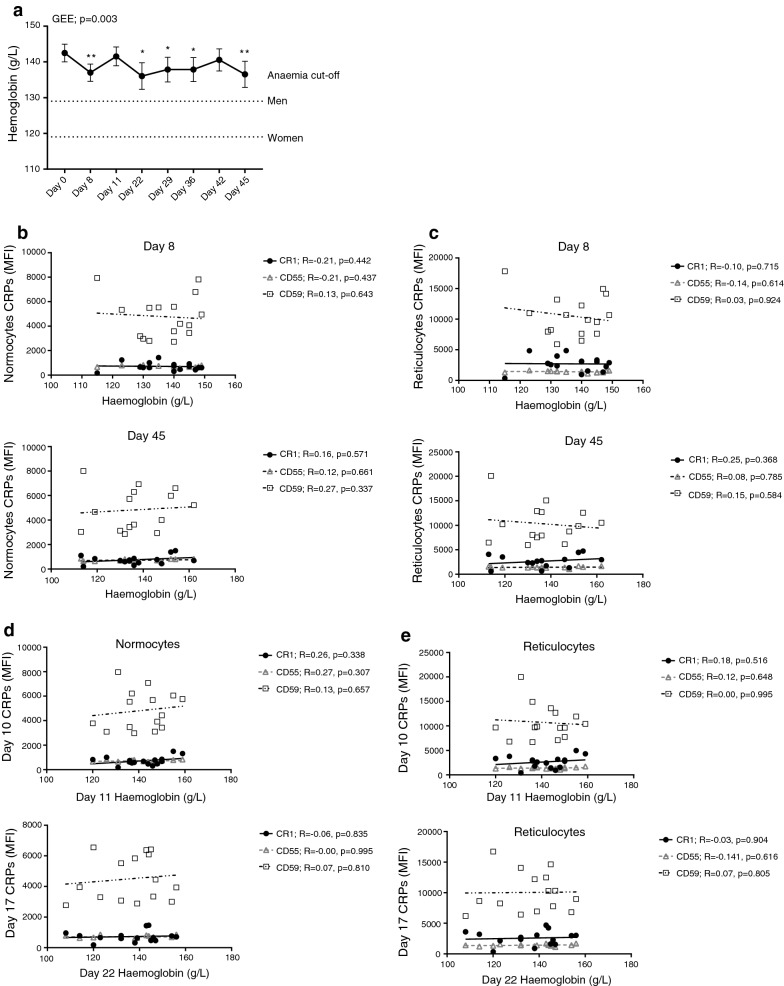
Relationship between changes to CRP and haemoglobin level in volunteers from IBSM trial. **a** Haemoglobin levels following induced blood stage malaria infection. Generalized estimating equation (GEE) and Wilcoxon signed-rank nonparametric tests between each group are indicated. *p < 0.05; **p < 0.01. Data are presented in connecting lines with mean values and error bars indicating standard error. Dotted lines across the graph indicate mild-anaemia threshold for men and non-pregnant women. Correlations between CRPs expression on normocytes (**b**) and reticulocytes (**c**) and haemoglobin levels at day 8 and 45 following inoculation. Correlations between CRPs expression on normocytes (**d**) and reticulocytes (**e**) at day 10 and 17 and haemoglobin levels at day 11 and 22 following inoculation. Spearman’s nonparametric correlation test is indicated

To understand the relationship between haemoglobin and CRPs, haemoglobin level was examined in volunteers participating in IBSM studies and this was correlated with CRP expression on RBC. Overall, there were only small changes observed to haemoglobin levels following IBSM. During primary *P. falciparum* infection, haemoglobin was reduced at all follow-up time-points compared to baseline (day 0), except for day 11 and 42 (Fig. 6a). However, these reductions were small, with only 5 individuals developing mildly-anaemia (cut-off: 129 g/L for men and 119 for women g/L) and none developing moderate anaemia (cut-off: 109 g/L for men and women). The fluctuations in haemoglobin during IBSM may be due the multiple and overlapping mechanisms mediating changes to haemoglobin during infection. For example, the drop in haemoglobin at day 8 (peak infection) maybe due to the rupture of infected RBCs at this timepoint, which then returns to normal following treatment, but then is reduced again due to other pathogenic mechanisms including CRP reduction, dys-erythropoiesis and others.

Due to differences in collection time-points for blood samples and haemoglobin measurements, matched CRP and haemoglobin data were only available for day 8 and 45; there was no significant correlation between CRP expression on normocytes or reticulocytes and haemoglobin level at these time points (Fig. 6b, c). To assess if early reduction in CRPs was associated with subsequent loss of haemoglobin, CRP expression was correlated with haemoglobin at later time-points. No significant correlations observed between CRP expression on normocytes or reticulocytes at day 10 with that of haemoglobin at day 11 nor CRP expression at day 17/18 with that of haemoglobin at day 22 (Fig. 6d, e). Taken together, the results found limited relationship between changes in CRPs and haemoglobin levels in volunteers participating in IBSM studies.

## Discussion

In the present study, the relationship between CRP expression and anaemia was investigated in two distinct human cohorts: (1) patients from a low malaria transmission region with uncomplicated falciparum and vivax malaria and mild anaemia; and, (2) volunteers undergoing *P. falciparum* IBSM. Consistent with previous studies demonstrating that CRP loss is associated with severe malarial anaemia [11, 19], the current results show that the expression of CRPs on RBC is also reduced in individuals with mild malarial anaemia associated with either falciparum or vivax malaria. Loss of CRPs is associated with haemoglobin [11, 19, 24, 25], suggesting that CRP loss may also be an important contributor to chronic malarial anaemia in low transmission settings. The present study also shows that CRPs are reduced on RBCs following experimental IBSM with *P. falciparum* where parasitaemia is very low. While there was no significant correlation in IBSM between reduced CRPs on RBC and subsequent changes to haemoglobin, patients in this model are treated very early following infection, well before they develop significant haemoglobin loss. Overall, data in this study show that CRPs are reduced on RBC even in mild malarial anaemia and also during low-density infections, supporting the hypothesis that CRP loss on RCB is a driver of malarial anaemia.

In Malaysian malaria patients, CRPs on normocytes and reticulocytes were reduced in patients with mildly anaemic *P. falciparum* and *P. vivax* infection, suggesting that the removal of CRPs is occurring during early stages of anaemia. While well-documented in severe malarial anaemia [19], significant loss of CRPs during mild malarial anaemia has not been fully described. Additionally, a positive association between CD59 expression and haemoglobin level was observed in this study. An in-vitro study using *P. falciparum*-infected RBCs demonstrated that parasitized RBCs were more resistant to complement-mediated lysis due to CD59 expression [26], suggesting its important role in protection from complement-mediated anaemia. The findings in this study also expand the evidence of a role for CRP-mediated anaemia pathogenesis to another geographical region in Southeast Asia, in a population with a different genetic background, suggesting this mechanism is independent of the genetic background of infected individuals. Due to limited number of samples available, some of the comparison of CRP expression may not necessarily be representative and statistically sufficient. Nevertheless, data in present study indicate that CRPs are removed in mild malarial anaemia, and is consistent with previous findings that suggest CRP removal is an important mechanism of malaria anaemia.

Expression of CR1, CD55 and CD59 on normocytes and reticulocytes were reduced in volunteers after *P. falciparum* infection. However, the magnitude of the loss of CR1 was greater on reticulocytes, suggesting that reticulocytes are more susceptible to complement-mediated attack compared to normocytes. Higher expression of CRPs on reticulocytes compared to normocytes has been described [19, 27–29], and the level of CR1 is directly correlated with the binding of circulating immune complexes [30, 31]. The mechanism of CRP removal is thought to occur through the formation of antigen-antibody immune complexes that binds to RBC CRPs, with the immune-complex/CRP then subsequently transported removed by macrophages [12, 15, 16, 32]. As such, the relatively higher CR1 expression on reticulocytes may result in reticulocytes binding more immune complexes during circulation, resulting in a larger loss of CR1 compared to normocytes.

The current study investigates longitudinal loss of CRPs for the first time and demonstrated that reduction of CRPs occurs even during low density *P. falciparum* infection prior to the onset of anaemia. This is consistent with a previous observation that shows complement activation is increased in volunteers with *P. falciparum* sporozoite induced infection [33]. Past clinical studies were not able to confirm this parasite driven loss of CRPs as the study samples were collected from cross-sectional enrolments of patients presenting with clinical malaria [11, 19, 20]. While the current data do not formally demonstrate a link between CRP loss and reduced haemoglobin levels and anaemia as there was no association between these two parameters in the IBSM cohort, data from the Malaysian study is supportive of a role of CRPs in driving anaemia. The pathogenic mechanism of malarial anaemia is complex and multifactorial [6, 9, 34]. Thus, it is likely that malarial anaemia is mediated by collective combinations of different pathogenic factors. Other major pathogenic mechanisms of malarial anaemia include dyserythropoiesis through bone marrow dysfunction [35, 36] and reduced deformability of both infected and uninfected RBC that is thought to cause splenic accumulation of RBCs [37, 38]. Volunteers from IBSM trial have very low parasite counts detected by qPCR by the time they are treated. Therefore, it is likely that patients are treated before this loss of CRPs from low density infections is insufficient to induce significant loss of RBCs from complement attack; indeed, the reduction of haemoglobin during experimental infection were very minor.

## Conclusions

This study shows that the expression of CRPs on normocytes and reticulocytes is reduced in mild anaemia in patients with falciparum and vivax malaria. Further, reduction of CRPs occurs early in infection, during low density parasitaemia and prior to the onset of anaemia. Taken together, the results demonstrate that removal of CRPs from RBCs during *Plasmodium* infection takes place early during infection and are consistent with CRP loss on RBCs as an important driver of the development of malarial anaemia.

## Data Availability

All data generated or analysed during this study are included in this published article.

## Abbreviations

RBC: red blood cell
CRP: complement regulatory protein
